# Successful Treatment with Intravenous Cyclophosphamide for Refractory Adult-Onset Still's Disease

**DOI:** 10.1155/2015/163952

**Published:** 2015-12-22

**Authors:** Yoshika Tsuji, Nozomi Iwanaga, Anna Adachi, Kinuyo Tsunozaki, Yasumori Izumi, Yuji Moriwaki, Kazuhiro Kurohama, Masahiro Ito, Atsushi Kawakami, Kiyoshi Migita

**Affiliations:** ^1^Department of General Internal Medicine and Rheumatology, Nagasaki Medical Center, Kubara 2-1001-1, Omura 856-8562, Japan; ^2^Nagasaki Medical Center, Kubara 2-1001-1, Omura 856-8562, Japan; ^3^Department of Hematology, Nagasaki Medical Center, Kubara 2-1001-1, Omura 856-8562, Japan; ^4^Department of Pathology, Nagasaki Medical Center, Kubara 2-1001-1, Omura 856-8562, Japan; ^5^Department of Rheumatology, Nagasaki University Hospital, Nagasaki 852-8121, Japan

## Abstract

We report a 64-year-old female case of intractable adult-onset Still's disease (AOSD). Initial high-dose steroid therapy combined with cyclosporin A was ineffective against macrophage-activation syndrome (MAS), which was accompanied by the systemic type of AOSD. Treatment for MAS with intravenous cyclophosphamide resulted in remission of AOSD and a reduction in the high doses of steroids. Efficacy of biologics against MAS in AOSD is unclear. Cyclophosphamide, a conventional cytotoxic agent, should be considered as one of the therapeutic options for refractory types of AOSD with MAS.

## 1. Introduction

Adult-onset Still's disease (AOSD) is a rare systemic inflammatory disorder of unknown etiology. AOSD is characterized by spiking fevers with an evanescent rash, arthritis, and multiorgan involvement [1]. According to the clinical presentation of this disease at diagnosis, two AOSD phenotypes may be distinguished: (i) a highly symptomatic, systemic, and feverish phenotype, which evolves into a systemic (mono- or polycyclic) pattern; (ii) a more indolent phenotype with arthritis, which evolves into a chronic articular pattern [2]. Steroid- or disease modified antirheumatic drugs-(DMARDs-) refractory AOSD cases currently benefit from recent insights into autoinflammatory disorders. Anticytokine treatment appears to be an efficient, well-tolerated, steroid-sparing treatment in systemic patterns [3]. Tocilizumab appears to be efficient in AOSD with active arthritis and systemic symptoms [4, 5]. Macrophage-activation syndrome (MAS) is a disorder characterized by hemophagocytosis and deregulation of T lymphocytes and macrophages and subsequent overproduction of cytokines [6]. MAS can occur in rheumatic disease and is frequently observed in patients with AOSD [7]. Despite major advances in the understanding of MAS, further studies are required to determine the therapeutic approach to regulate the “cytokine storm.” Cytokine-directed therapies have the potential to target the effector molecules in MAS [8] and MAS complicating AOSD were successfully treated by these therapies [9, 10]. However, manifestations of MAS during biologics therapy have been also reported [11]. We report a case of MAS that was complicated by AOSD and was successfully treated with intravenous cyclophosphamide (IV-CY).

## 2. Case Report

A 64-year-old Japanese woman was admitted to our hospital because of fever, polymyalgia, and sore throat. She had been well until approximately 2 weeks earlier, when her sore throat developed. One week later, she developed a spiking fever and an evanescent skin rash on the upper limbs and trunk, followed by polyarthralgia. Upon examination, inflammatory arthritis of the shoulder and elbow joints and muscle tenderness of the upper extremities coinciding with the erythematous skin rash on her trunk and spiking fever (>39°C) were evident.

Initial blood data on admission were as follows (Table 1): leukocytes, 26,000/*μ*L (neutrophils, 90.0%); hemoglobin, 12.0 g/dL; platelets, 29.3 × 10^4^/*μ*L; erythrocyte sedimentation rate, 71 mm/h; prothrombin time, INR 1.17; fibrinogen, 684.2 mg/dL; FDP, 13.2 *μ*g/mL; C-reactive protein, 9.95 mg/dL; soluble interleukin-2 receptor, 1850 IU/L; and ferritin, 11740 ng/mL. Anti-nuclear antibodies and anti-CCP antibodies were negative. Serological tests for Epstein-Barr virus (EBV) and cytomegalovirus (CMV) antigenemia showed negative results. Computed tomography (whole body) and fiberscopic analysis (upper and lower intestine) did not show the findings suggestive for malignancies. The diagnosis of AOSD was made according to Yamaguchi's diagnostic criteria [12] based on the above-mentioned findings, including spiking fever, polyarthritis, salmon pink skin rash, leukocytosis, sore throat, and elevated serum transaminases. These findings met the classification for the systemic type of AOSD.

**Figure 1:**
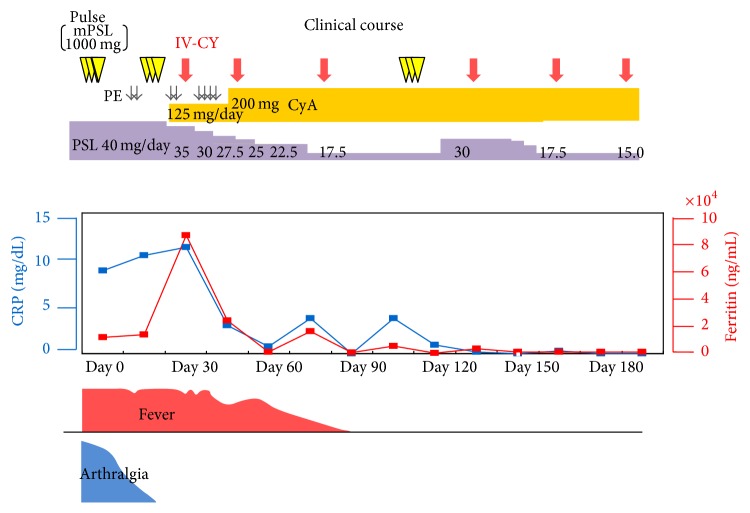
Clinical course. CyA: cyclosporine A; IV-CY: intravenous cyclophosphamide; mPSL: methylprednisolone; PE: plasma exchange.

Steroid pulse therapy (methylprednisolone 1000 mg for 3 days) was started and followed by oral prednisolone (40 mg daily), which was combined with oral cyclosporin A (200 mg/day). After a transient improvement, a disease flare, which included spiking fever and further elevated levels of serum transaminases and ferritin, occurred on day 13 (Figure 1). On day 30, the white blood cells counts decreased (21000/*μ*L to 9200/*μ*L) and mild thrombocytopenia (36.7 × 10^4^/*μ*L to 13.7 × 10^4^/*μ*L) appeared within 10-day duration. Additionally, fibrinogen (687.5 mg/dL to 349.2 mg/dL) was decreased, and conversely triglyceride (186 mg/dL to 235 mg/dL) and FDP (7.4 *μ*g/dL to 49.7 *μ*g/dL) were increased. These subtle laboratory alternations also support the association of MAS.

**Figure 2:**
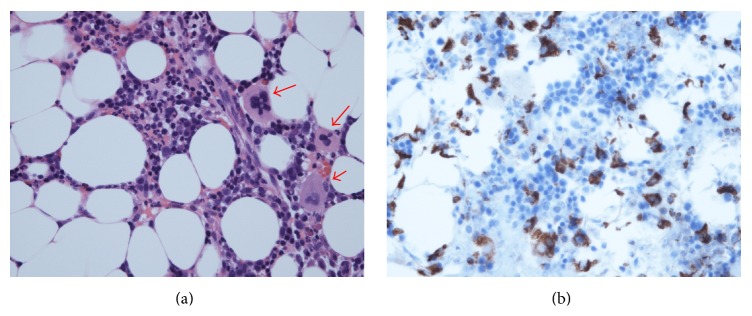
(a) The phagocytic reticular cells (arrows). The phagocytosis of mature neutrophils by phagocytic reticular cells is present. Hematoxylin and eosin (HE) staining. (b) Immunohistochemistry showing monocyte infiltrations in bone marrow. The CD68^+^ cells increased and were diffusely distributed in stroma of bone marrow. Immunohistochemical staining using antibodies against CD68.

Repeated serological analysis indicated no infection with parvovirus B19, hepatitis B virus (HBV), HCV, and EBV, and CMV antigenemia was not detected. Bone marrow aspiration showed phagocytosed blood cells and massive infiltrated CD68-positive monocytosis (Figure 2). Based on the negative results for infectious agents, these observations indicated that the patient had hypercytokinemia and MAS associated with AOSD. She was treated with plasma exchange followed by steroid pulse therapy. Despite these intensified treatments, the spiking fever was sustained, and this was accompanied by sustained elevation of serum ferritin levels. Since the cytopenia was mild, we selected the low dose (500 mg) IV-CY treatment for MAS. She was treated with IV-CY (500 mg), and these combination therapies effectively reduced the disease activity of AOSD and MAS (Figure 1). Thereafter, the patient has been successfully maintained on IV-CY every one-month interval (total of six times) in combination with a maintenance dose of oral prednisolone and oral cyclosporin A.

## 3. Discussion

AOSD often poses a diagnostic and therapeutic challenge, and clinical guidelines are lacking [13]. MAS is a disorder that is characterized by hemophagocytosis, inappropriate systemic proliferation of histiocytes throughout the reticuloendothelial system, dysregulation of T lymphocytes and macrophages, and subsequent overproduction of cytokines, such as interleukin-1, interleukin-6, and interferon-*γ* [14]. Among the rheumatic diseases, sJIA and AOSD are often associated with MAS, and one retrospective study indicated a high frequency (12%) of MAS in AOSD patients [15].

The application of new biological agents may provide clinicians with useful tools for the management of this complex systemic disorder [16]. In patients with systemic involvement with or without articular manifestation, biologics, such as interleukin-1 inhibitors and tocilizumab, have favorable effects in AOSD [17]. A growing body of evidence suggests the efficacy of biological agents against the treatment of steroid- or DMARDs-refractory AOSD [18]. However, these reports on AOSD did not include cases with hemophagocytic syndrome or MAS. Therefore, the efficacy of tocilizumab therapy for the active phase of AOSD with MAS is unclear. Physicians should be cautious of possible induction of biologics-related MAS [19, 20].

In a study by Wouters et al., of 18 trials of antirheumatic drugs used in 45 patients with disease refractory to NSAIDs and corticosteroids, only eight (44%) proved clinically efficacious [1]. Second-line treatments for patients with steroid-resistant refractory AOSD include cyclosporin A, leflunomide, and cyclophosphamide [21]. Studies have suggested that the use of immunosuppressive agents should be reserved for cases in which the combination of antirheumatic drugs and steroids fails or when a reduction in the requirement for steroids is desired, owing to either lack of tolerance or adverse events [21]. Immunosuppressive agents that have been studied include cyclosporin A, azathioprine, and cyclophosphamide with a modest success and overall response across studies of approximately 40% [22]. Among these immunosuppressive treatments, the efficacy of IV-CY has been shown to be superior to that of cyclosporine A in autoimmune-associated hemophagocytic syndrome [23]. Because CYC is a potent immunosuppressive treatment for refractory autoimmune diseases, IV-CY might be able to be used to treat patients with refractory AOSD, as well as AOSD-associated MAS. Based on the observations made in our case, complete suppression of AOSD by IV-CY therapy in combination with cyclosporin A may be a therapeutic strategy for the systemic type of AOSD with MAS. In the hemophagocytic lymphohistiocytosis (HLH) treatment protocol by the International Histiocyte Society the combination of steroids, cyclosporine A, and cytotoxic drug forms the major treatments for MAS [24]. Therefore, it is possible that the efficacy of IV-CY could be drawn by the combination with steroids and cyclosporine A, which were used in the present case.

The number of case reports on autoimmune-associated MAS is increasing. However, treatment efficacy and outcomes in AOSD with this complication are not well understood. IV-CY can be a strong second-line treatment for refractory AOSD showing activation of macrophages. This treatment allows a large reduction in corticosteroid doses. This is beneficial to patients who are resistant to high doses of corticosteroid and may be a reasonable strategy for treating AOSD with MAS.

## Figures and Tables

**Table 1:** Laboratory findings on admission.

Peripheral blood	
Red blood cells	382 × 10^6^/*μ*L
Hemoglobin	12.0 g/dL
Hematocrit	33.9%
White blood cells	26,000/*μ*L
Neutrophil	90.0%
Monocyte	3.0%
Lymphocyte	5.0%
Platelet	29.3 × 10^4^/*μ*L
Blood chemistry	
Total protein	5.7 g/dL
Total bilirubin	1.0 mg/dL
AST	34 IU/L (7−33)
ALT	22 IU/L (5−30)
Lactate dehydrogenase	1095 IU/L (119−229)
Alkaline phosphatase	611 IU/L (80−250)
Gamma-glutamyl transpeptidase	257 IU/L (5−55)
Creatinine kinase	14 IU/L (60−160)
Total cholesterol	186 mg/dL
Triglyceride	225 mg/dL
Blood urea nitrogen	13.6 mg/dL
Creatinine	0.4 mg/dL
Alb	2.1 g/dL
Na	131 mEq/L
K	4.1 mEq/L
Cl	91 mEq/L
Serological tests	
C-reactive protein	9.95 mg/dL (<30)
Erythrocyte sedimentation rate	71.0 mm/hr
Ferritin	11740 ng/mL (<170)
C3	142 mg/dL (86–160)
C4	26 mg/dL (17–45)
ANA	(—) (<40)
Anti-CCP Ab	<0.6 U/mL (<4.5)
MPO-ANCA	<1.0 U/mL
RR3-ANCA	<1.0 U/mL
IgG	1340 mg/dL (900–2000)
IgM	108 mg/dL
IgA	338 mg/dL
sIL-2R	1850 U/mL
Coagulation	
PT time (INR)	67.8% (1.19)
fibrinogen	684.2 mg/dL
FDP	13.2 *μ*g/mL
Microbiological test	
HCV-Ab	(—)
HBsAg	(—)
CMV antigenemia	(—)
EBV-EBNA	×40
EBV-VCA IgM	<×10
EBV-VCA IgG	×160
Blood culture	(—)
*β*-D-Glucan	<3.4 pg/mL
Urinalysis	Normal

ANA: anti-nuclear antibody; ANCA: antineutrophil cytoplasmic antibody; CMV: cytomegalovirus; EBV EBNA: Epstein-Barr virus, nuclear antigen; EBV-VCA: Epstein-Barr virus viral-capsid antigen; HBsAg: hepatitis B surface antigen; HCV: hepatitis C virus; MMP-3: matrix metalloproteinase-3; MPO: myeloperoxidase; RF: rheumatoid factor; RR3: proteinase 3.
